# Bridging the divide: preclinical research discrepancies between triple-negative breast cancer cell lines and patient tumors

**DOI:** 10.18632/oncotarget.22916

**Published:** 2017-12-04

**Authors:** Andrew Sulaiman, Lisheng Wang

**Affiliations:** ^1^ Department of Biochemistry, Microbiology and Immunology, Faculty of Medicine, University of Ottawa, Ottawa, Ontario K1H 8M5, Canada; ^2^ China-Canada Centre of Research for Digestive Diseases, University of Ottawa, Ottawa, Ontario K1H 8M5, Canada; ^3^ Institute of Digestive Diseases, Longhua Hospital, Shanghai University of Traditional Chinese Medicine, Shanghai 201203, China; ^4^ Regenerative Medicine Program, Ottawa Hospital Research Institute, Ottawa, Ontario K1H 8L6, Canada; ^5^ Ottawa Institute of Systems Biology, University of Ottawa, Ottawa, Ontario K1H 8M5, Canada

**Keywords:** triple-negative breast cancer, cell lines, patient derived xenograft, translational research

## Abstract

Triple-negative breast cancer (TNBC) is the most refractory subtype of breast cancer and disproportionately accounts for the majority of breast cancer related deaths. Effective treatment of this disease remains an unmet medical need. Over the past several decades, TNBC cell lines have been used as the foundation for drug development and disease modeling. However, ever-mounting research demonstrates striking differences between cell lines and clinical TNBC tumors, disconnecting bench research and actual clinical responses. In this review, we discuss the limitations of cell lines and the importance of using patients’ tumors for translational research, and highlight the usage of patient-derived xenograft (PDXs) models that have emerged as a clinically relevant platform for preclinical studies. PDX tumors possess tumor heterogeneity with similar cellular, molecular, genetic and epigenetic properties akin to those found within patients’ tumors. Moreover, PDX and clinical tumors possess abnormal vasculature with higher blood vessel permeability, a feature that is not always demonstrated in *in vivo* cell line xenografts. Development of clinically relevant, novel drug-nanoparticles capable of accumulating in PDX tumors through the enhanced permeability and retention effect in tumor vasculature may lead to new and effective TNBC treatments.

## INTRODUCTION

Breast cancer remains a leading cause of death in women throughout the world. Triple negative breast cancer (TNBC) accounts for only 15-20% of all breast cancer, but is disproportionally associated with the majority of breast cancer related deaths [1]. Chemotherapy is currently the mainstay of systemic medical treatment for TNBC. However, it is associated with severe off-target tissue toxicity, rapid drug-resistance, and enrichment of cancer stem cells [2, 3]. As such, development of targeted therapies for TNBC is an unmet medical need.

Over the past several decades, *in vitro* and *in vivo* preclinical research commonly uses over 27 TNBC cancer cell lines to study cancer pathogenesis, disease advancement, and drug effectiveness. However, a growing disconnection between results generated using TNBC cell lines and clinical trials has been observed. A recent example is the *in vitro* and *in vivo* results of PARP inhibitor veliparib. Veliparib is an oral inhibitor of Poly (ADP-Ribose) Polymerase (PARP) 1 and 2, which enhances the activity of DNA damaging agents in DNA repair to promote apoptosis. *In vitro*, veliparib is capable of suppressing the expression of Snail which promotes epithelial to mesenchymal transition, tumor metastasis and drug resistance. It also sensitizes the MDA-MB-231 TNBC cell line to chemotherapeutic drug doxorubicin, resulting in increased apoptosis [4]. *In vivo*, veliparib sensitizes MDA-MB-231 tumors to TMZ (temozolomide, an alkylating agent) in a SCID (severe combined immune deficiency) mouse model [5]. The effectiveness of other therapeutic combinations with veliparib has also been demonstrated *in vivo* xenograft mouse models using cancer cell lines [5, 6].

Clinical trials, however, failed to demonstrate the efficacy of veliparib in combination with a DNA damaging agent for the treatment of breast cancer including TNBC. The phase II clinical trial (NCT01506609) recruited 193 metastatic breast cancer patients treated with either the placebo or veliparib in a combination of carboplatin and paclitaxel. Progression-free survival in the control group (chemotherapeutic drugs alone) was 12.3 (9.3–14.5) months compared to the 14.1 (11.5–16.2) months in the combination group, showing statistically insignificant difference (*p* value = 0.231) [7, 8]. Overall survival in the control was 25.0 (18.1–34.8) months and the combination of veliparib and chemotherapy was 28.5 (22.4– not reported results), which was insignificant (*p* value = 0.148) [8]. Despite these results, veliparib in combination with paclitaxel and carboplatin followed by doxorubicin and cyclophosphamide advanced into phase III clinical trials (NCT02032277) for the treatment of TNBC [9]. 634 TNBC patients were involved in the study and treated with veliparib or placebo in combination with paclitaxel and carboplatin followed by doxorubicin and cyclophosphamide. There was no significant difference in the efficacy of treatment (53.2% veliparib + chemotherapy *vs* 57.5% placebo + chemotherapy, *p* = 0.36) [9]. This recent failure is by no means a rarity as many similar results have been reported [10–14]. This highlights the disconnection between cell lines *in vitro* and *in vivo* preclinical research and human clinical trials. The translational disparity led to the US National Cancer Institute halting the usage of 60 human cancer cell lines for drug-screening in 2016 and recommending to use patient derived xenograft (PDX) for future research. Appropriate models used in preclinical/translational studies may bridge the divide [15]. In this regard, PDXs have shone as clinically relevant models in comparison to breast cancer cell lines due to their ability to better represent the original tumor's biology and retain the original tumor's architecture and organization [16].

## THE LIMITATIONS OF CELL LINES IN PRECLINICAL RESEARCH

Breast cancer cell lines used for conventional analysis were originally harvested and generated from patient tumor samples after *in vitro* culture for years or decades. The deviancies observed are thought to arise through selection of specific populations and changes over time to promote adaption to artificial culture environments. Breast cancer cell lines are capable of growing indefinitely and undergoing freezing-thawing cycles for several decades. It has been demonstrated that breast cancer cell lines possess a moderately high mutation frequency in comparison to patient tumors. Over many *in vitro* passages, these mutations can accumulate, possibly making the cells differ dramatically from their starting source [17–20]. Additionally, these mutations can promote certain traits which provide a survival benefit for *in vitro* growth in a plastic dish. This would promote clonal selection for the fittest subpopulations [21]. Continuous propagation of cells in a petri dish would also result in accumulating epigenetic alterations [22]. It has been demonstrated that human cancer cell lines possess altered methylation patterns after culture [23]. Altered DNA methylation affects gene and protein expressions, subsequently impacting signal pathways and therapeutic responses. Additional reports have shown that DNA methylation differs dramatically between cancer cell lines in comparison to patient tumors, making epigenetic studies using cell lines discordant with clinical settings [24, 25].

One example was the expression of ER/PR/HER-2 receptors in two TNBC tumors obtained from patients and cultured for 150 passages. Originally, these receptors were all absent in the primary tumors harvested from the TNBC patients [26]. Miller *et al* also recently showed that there were almost no overlaps in gene expression between glioblastoma samples grown in mice and cultured on a dish after 2-3 weeks, suggesting a marked modification of tumor biological features after short-term culture in petri dishes [27].

**Figure 1:**
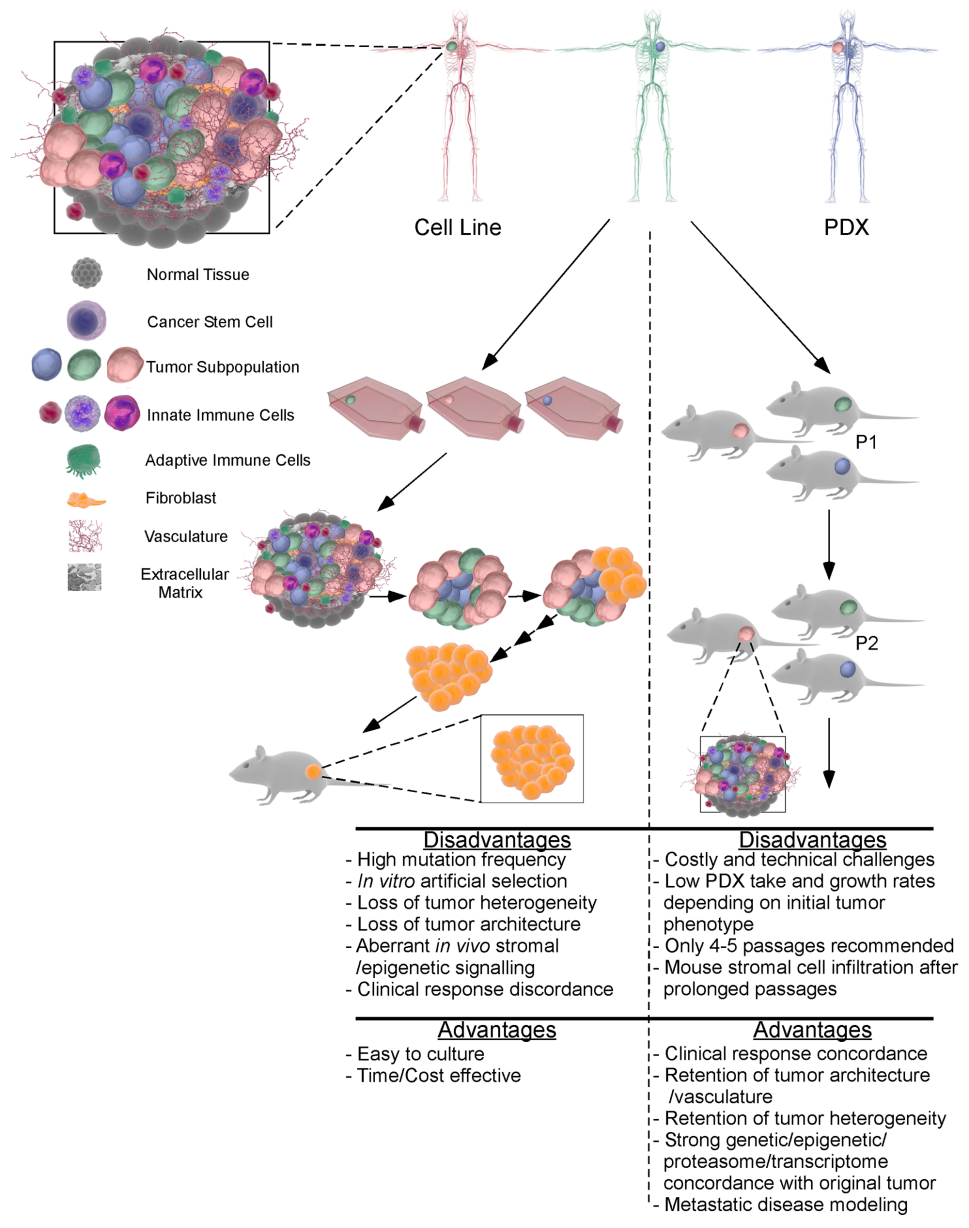
The main differences between PDX and cell line xenografts for preclinical research *In vitro* culture of patient samples leads to a loss of tumor architecture and heterogeneity. The resultant adherent tumor cells are subject to culture selection and adaptation to artificial conditions, leading to the generation of a cell line from a subpopulation of the original patient's tumor containing multiple cell types and subpopulations. Subsequent *in vitro* and *in vivo* experiments preformed using a subpopulation of cells may result in discrepancy between breast cell lines and clinical observations and clinical trials. In contrast, implanting breast tumor immediately after harvesting from patients into an immune deficient mouse model can preserve tumor heterogeneity, architecture and stromal and extracellular components. After *in vivo* expansion, the PDX tumors retain original tumor properties for up to 4-5 passages. In sharp contrast to cancer cell line xenografts, drug responses of PDX models are consistent with patients, making PDX model an invaluable tool for translational research.

This dramatic deviance is largely associated with the disruption of the original tumor structure and microenvironment which is comprised of a heterozygous mixture of different subpopulations of tumor cells, macrophages, fibroblasts, endothelial cells, stromal cells, the extracellular matrix, etc. [28, 29]. Cancer cell lines do not represent these heterozygous components. Rather, during initial harvesting and culturing, subpopulations adapted better for *in vitro* petri dish environment (e.g. cancer associated fibroblast cells) are commonly selected for, overtaking the other tumor cells and resulting in a relative homozygous population overtime [30–32]. Culture methodologies which inhibit fibroblastic growth and promote epithelial proliferation, still fall victim to one dominant tumor subpopulation [31, 33]. This artificial selection makes the therapies developed highly effective on a particular cell subtype rather than the whole heterozygous tumor and its extracellular matrix and tumor microenvironment, which disconnects the bench results from the clinical trials. *In vivo* studies, human breast cancer cell lines are commonly mixed with matrigel and injected into mouse mammary pad to resemble the clinical settings. However, in addition to the aforementioned limitations, this sudden influx of cancerous cells bypasses the early development of a tumor in the patient and skips over the formation of the tumor microenvironment [34, 35]. This may in part, explain the divergence between the high frequency of bone metastasis for patient with breast cancer (~70% of all breast metastasis) and the very low frequency of spontaneous metastasis of breast cancer cell line implanted in the mammary fat pad [36–38]. As such, to mimic bone metastasis, breast cancer cell lines must be injected either *via* intracardiac, tail vein or intra-osseous, or specialized cell lines must be utilized [38–41]. Additionally, the monocultured breast cancer cell lines do not include factors commonly dysregulated in the tumor such as hypoxia, inflammation, vascularity, stromal cells, immune cell infiltration, and aberrant signalling pathways [42]. These factors work together to regulate tumor microenvironment, tumor growth and metastasis. As such, translatable research requires a breast cancer model freshly isolated from the patient without disturbing tumor structures to encompass all of these factors and retain tumor heterogeneity and microenvironment (Figure 1).

## THE IMPORTANCE OF USING PATIENTS TUMORS AS MODELS FOR PRECLINCIAL RESEARCH

Considerable observations obtained from patients’ tumors cannot be mimicked by using breast cancer cell lines. Acerbi *et al* recently demonstrated that crosstalk between the extracellular matrix and inflammation promotes invasion in 20 breast cancer patient biopsies [43]. Increased amounts of collagen were deposited within invasive breast cancer. Furthermore, the collagen was thicker, underwent a linear reorganization in the stroma of the invasive lesions, and was associated with increased mechano-signalling and increased stromal stiffness. The invasive edge of the tumors possessed the greatest stromal stiffness illustrating regional stromal heterogeneity. This stiffness at the tumor edge was caused by accumulating activated macrophages and increased TGF-β activity, suggesting a crosstalk between macrophage accumulation, stromal stiffness and tumor invasion. TNBC patient tumors possessed the greatest stromal stiffness, macrophage accumulation, and TGF-β activation at the tumor front compared to the other breast cancer subtypes. Additionally, TNBC exhibited increased YAP (Yes-associated protein) signalling that correlated with stromal stiffness, tumor aggression and invasion. YAP is a mechanically activated signaling pathway that is associated with cancer stem cells (CSCs) and poor patient prognosis [44–46]. This study highlights the multifaceted interplay between tumor cells, the extra cellular matrix and the immune system, which cannot be modeled by the cultured breast cancer cell lines and their xenografts.

Using patients’ tumor samples, Liu *et al*, demonstrated that there exist two pools of CSCs within the breast cancer. A mesenchymal, migratory CD44^+^/CD24^-^ CSC subpopulation exists at the tumor edge, while an epithelial, proliferative ALDH^+^ CSC subpopulation resides within the tumor core. Moreover, interconversion (plasticity) between the fractionated two CSC subpopulations was observed, and both epithelial and mesenchymal CSCs were responsible for metastasis and tumor reconstitution at a secondary location. Controversially, *in vivo* xenograft analyses of breast cancer cell lines were unable to demonstrate ALDH^+^ or CD44^+^/CD24^-^ CSC localization patterns, or demonstrate a correlation between the frequency of CD44^+^/CD24^-^ CSCs and tumor metastasis as observed in patients with breast cancer [47, 48].

Recent reports demonstrated that a hybrid epithelial/mesenchymal CD44^+^/CD24^-^/ALDH^+^ CSC subpopulation is more tumorigenic then its pure counterpart, although its role in metastasis and secondary tumor formation remains to be elaborated [49–51]. Using patients’ metastatic breast cancer pleural effusions, Shiraishi *et al* demonstrated that CD44^+^/CD24^-^/ALDH^+^ CSCs possessed a greater hypoxic response to hypoxia inducible factor (HIF-1α) signalling [49]. This response in turn promoted an epithelial to mesenchymal transition through the inhibition of E-cadherin and stimulation of Notch-1, Jagged-1, TGF-β, Slug and Snail, which enhanced metastasis and secondary tumor formation *in vivo*. Interestingly, CD44^+^/CD24^-^/ALDH^-^ CSCs in contrast, did not undergo EMT upon hypoxia. Instead, hypoxia induced HIF-1α to bind directly to the ALDH1A1 promoter, which converted CD44^+^/CD24^-^ /ALDH^-^ CSCs into CD44^+^/CD24^-^ ALDH^+^ CSCs. The newly converted ALDH^+^ cells expressed angiogenic genes rather than EMT-related genes and were able to generate pulmonary metastasis [49]. In comparison to patient tumors, breast cancer cell lines differentially expressed ALDH, CD44 and/or CD24, making interpretation of experimental results difficult [52]. These studies further highlight the importance of using patients’ tumor samples over breast cancer cell lines for the studies of inter/intra tumor interactions, CSC localization and plasticity, tumor heterogeneity and metastasis in translational medicine [47, 53–55].

## PATIENT-DERIVED XENOGRAFT MODELS

While fresh patients’ tumors are a great model for cancer research, their availability, quantity and quality are limiting factors for widespread usage [56]. Patient-derived xenograft (PDX) models become an excellent alternative and are readily available for researchers. PDX models are generated through the transplantation of patients’ tumor tissues into an immunocompromised mouse [57]. The implanted tumors are expanded and serially passaged in mice. PDX procedures exclude tissue dissociation and *in vitro* culture, which prevents cell adaptation to artificial culture system, clonal selection, and homogeneity (Figure 1) [58].

Another advantage of the PDX model over cell lines is the preservation of the original tumor architecture and organization such as vasculature and stromal components [16]. This is thought to represent the original tumor's biology and retain the interactions between the tumor and its microenvironment [16, 59]. PDX models also retain intra/inter-tumor heterogeneity, gene expression, single nucleotide polymorphisms, copy number variants and chromosomal architecture of the original tumors [16, 58–63].

The ability of the PDX models to simulate the original patients’ tumors may explain the strong correlation between PDX models and actual patient responses [64–67]. Zhang *et al* demonstrated this through implanting a series of human breast tumor tissues into the mammary fat pad of immunodeficient mice [68]. The tumor growth was correlated with tumor grade and the absence of estrogen (ER)/progesterone (PR) expression. After successful engraftment and growth, it was found that all PDXs retained the primary tumors’ histologic phenotypes. PDXs were also evaluated at the transcriptome, proteasome, and genome levels across multiple generations, and all closely resembling the original tumors [68]. Moreover, in a close resemblance to actual breast cancer progression, 48% of PDX tumors exhibited pulmonary metastasis after implantation into mammary fat pad. More importantly, clinical relevance was compared by assessing PDX response to the same treatment regime that had been used in the same patients giving rise to the PDX. Of 13 PDX tumors, 12 (92%) showed the same response as did patients to the chemotherapeutic drugs such as doxorubicin, paclitaxel or dasatinib amongst others, illustrating a high correlation between patients and PDX models [68].

In another report, Marangoni *et al* implanted 200 breast adenocarcinoma samples into the fat pad of athymic mice and stably generated 22 PDXs. They demonstrated that high breast grade tumors were superior to lower grade counterparts for engraftment and growth. Again, the original patient tumor histology, genomic rearrangements, chromosomal amplifications, and gene expression profiles were preserved in PDXs. Spontaneous metastasis was observed in 10/22 PDXs (45%), which also exhibited similar histology to the original tumors. Similar responses to chemotherapy (e.g. docetaxel/5-flurouracil/trastuzumab) between patients and their PDXs were also demonstrated in five out of seven cases [69, 70].

PDX models also retain the epigenetic patterns of the original patient tumor. Guilhamon *et al* demonstrated that in osteosarcoma and colon cancer, methylation profiles of PDXs were well preserved compared to the primary patient tumor with only 2.7% of CpG sites undergoing a major methylation shift in PDXs [71]. The second passage of PDXs showed only 0.07% of alternations in CpG methylation sites in comparison to the first passage [71]. Tomar *et al* also demonstrated that only 0.66-1.17% of CpGs were significantly altered after 3 passages compared to the original patient tumor in high-grade serious ovarian cancer PDXs [72]. While chemotherapy did not alter the DNA methylation pattern, treatment with decitabine (a demethylation agent) significantly demethylated 10.6% CpG sites and inhibited *in vivo* PDX tumor growth. Together, these studies suggest the epigenetic stability of PDX models and their suitability for epigenetic studies in comparison to cancer cells lines [72].

Short-term *ex vivo* cultured PDXs have also been used for pre-clinical high-throughput drug screening. Bruma *et al* showed that all PDX tumor tissues they tested could be successfully cultured *ex vivo* for a short period (n=27). These short-term *ex vivo* cultured PDX tissues retained tissue architecture, molecular and genetic features of *in vivo* PDXs. Of 40 *ex vivo* cultured PDX tissues used for drug screening, 33 (82.5%) were verified by *in vivo* PDX models, suggesting that *ex vivo* cultured PDX tissues can be used for high-throughput preclinical drug screening [73].

The predictive power of PDX models has led to the development of co-clinical trials, where patients and mice implanted with PDX tumors developed from the patient will be treated simultaneously or retrospectively. This allows for validation of the PDX results generated, and determination of factors affecting drug response/efficacy/resistance [74]. These personalized approaches are currently being investigated for various cancer types in multiple ongoing clinical trials [75]. One particular ongoing co-clinical trial for the treatment of TNBC is to study the effects of neoadjuvant docetaxel in combination with carboplatin in patients with stage 2-3 TNBC who have not achieved a pathologic complete response due to chemotherapeutic resistance (NCT02124902) [76]. The PDX models in this study will be developed simultaneously to determine chemotherapeutic response between patients, PDX take rates and to identify signatures of chemotherapy resistance and response [76]. Table 1 summarizes current active clinical trials using both PDX models and patients, investigating mechanisms underlying tumor progression, metastasis, and drug response and resistance.

**Table 1:** List of ongoing clinical trials using PDX models

Rank	NCT number	Title	Recruitment	Conditions
1	NCT03164863	Onco4D(TM) Biodynamic Chemotherapy Selection for Breast Cancer Patients	Recruiting	Breast Cancer
2	NCT02752893	Estrogen Receptor-Positive Breast Cancer Patient-Derived Xenografts	Recruiting	Breast Cancer
3	NCT02732860	Personalized Patient Derived Xenograft (pPDX) Modeling to Test Drug Response in Matching Host	Enrolling by invitation	Colorectal Neoplasms|Colorectal Cancer|Breast Cancer|Breast Neoplasms
4	NCT02455882	Tissue Procurement Protocol for Patients Undergoing Treatment for Early-Stage Breast Cancer	Recruiting	Breast Cancer
5	NCT02315196	Pegylated Liposomal Doxorubicin Hydrochloride and Carboplatin Followed by Surgery and Paclitaxel in Treating Patients With Triple Negative Stage II-III Breast Cancer	Recruiting	Estrogen Receptor-negative BreastCancer|HER2-negative BreastCancer|Progesterone Receptor-negativeBreast Cancer|Stage IIA BreastCancer|Stage IIB Breast Cancer|Stage IIIABreast Cancer|Stage IIIB Breast Cancer|Stage IIICBreast Cancer|Triple-negative Breast Cancer
6	NCT02247037	Patient-derived Xenograft (PDX) Modeling of Treatment Response for Triple Negative Breast Cancer	Recruiting	Triple Negative Breast Cancer
7	NCT02124902	Neoadjuvant Treatment of Triple Negative Breast Cancer Patients With Docetaxel and Carboplatin to Assess Anti-tumor Activity	Recruiting	Triple Negative Breast Neoplasms

Despite these advantages, PDX models are not perfect (Advantages/Disadvantages being summarized in Figure 1). The growth rate of PDX models are very slow compared to cell culture and xenografts generated using cancer cell lines. PDX implanted will take around 4-8 months for the development of a preclinical research specimen [74, 77]. Low engraftment rate persists as a critical challenge for PDX models. It was reported that TNBC possessed 53.8% of engraftment compared to 15.6% for hormone receptor positive breast cancer [78]. However, the established PDX samples exhibit over 90% engraftment rate despite low success for the primary PDX. The considerable established PDX samples that have been well characterized are currently available from research institutes or companies. The growth rate in each PDX mouse can also be highly variable depending on the quality and location of the tissues prepared from the same tumor.

Additionally, passaging the PDX samples in mice requires more resources, time and expertise in comparison to cell lines. Long-term passaging of PDX samples also affects PDX characteristics. Pearson *et al* demonstrated that PDXs of human head and neck squamous cell carcinoma increased their growth rate and displayed histopathological features of a higher tumor grade after prolonged *in vivo* passages [79]. To avoid deviations, it is recommended to use low passages (less than 5 passages). McAuliffe *et al* showed that high passages of breast cancer PDX exhibited some aberrations in P13K/mTOR signalling, an abrupt loss of human DNA in the PDX tumor and an increase in murine DNA. This was followed by the spontaneous generation of murine mammary adenocarcinoma [78]. Additionally, after 3-5 passages, the tumor stroma has been found to be replaced by the host mouse stroma which could influence stromal signalling, tumor rigidity, macrophage infiltration, autocrine and paracrine signalling, possibly deviating PDX from the original patients’ tumors [74].

Another limitation for current PDX models and for cancer cell line-xenografts is the requirement for the tumor to be implanted into an immunodeficient mouse for tumor engraftment and growth. Due to the lack of an immune system, the PDX model is not practical for immunological research. New PDX models have been proposed to address these issues by humanizing the immune deficient mice (e.g. JAX NSG). The human immune system will be generated through early transplantation of human hematopoietic stem cells into immunodeficient mice, followed by PDX implantation. This model will allow for assessment of immuno-tumor interactions in PDX [80]. This advancement can finally allow for the studies of human chimeric antigen receptor T cell, anti-PDL/PDL-1 and CTLA-4 in a PDX model.

## PDX MODELS AND NANOMEDICINE

Different from normal vascular system, the presence of endothelial gaps and transcellular holes in tumors increases blood vessel leakiness [81]. It is also found that tumor vasculature lacks vascular hierarchy, and possesses architectural abnormalities (heterogeneous, disorganized, branched/overlapped, and/or loosely connected) that resist blood flow and promote the extravascular erythrocyte accumulation (blood lakes) [81–84]. This in turn promotes improper nutrient translocation to the tumor and insufficient metabolite clearance, resulting in ischemia, hypoxia, acidic tumor environment, and necrosis. Increased HIF-1 in the tumor further enhances abnormal angiogenesis and tumor growth [82, 85].

PDX models have been demonstrated to be capable of representing human tumor angiogenesis [62]. The tumor vasculature comprised of human endothelial cells has been shown to mirror the donor patients’ tumor angiogenesis up to 35 days after implantation [56, 86]. In contrast, cancer cell line xenografts exhibit different vasculature from patients’ tumors, leading to contradictory effectiveness in angiogenic therapy [87–89]. Since angiogenesis is not only regulated by human tumor cells but also by human stromal cells and extracellular matrix, this might be a possible cause for the discrepancy as cancer cell line xenografts lack the components of human stromal cells and human extracellular matrix [90–92].

Abnormal tumor vasculature plays a key role in nanoparticle-based therapy. Nanotechnology applications in cancer have revolutionized the landscape of cancer drug development by their uniquely appealing features, such as improved blood circulation, higher tumor accumulation and reduced toxicities leading to a higher therapeutic index. Upon systemic administration, therapeutic nanoparticles have been shown to accumulate in tumors as a result of a multitude of biological processes involving mainly leaky tumor vasculature, poor lymphatic drainage and other minor events as well as enhanced permeability, and retention properties of the nanoparticle itself [93–98]. As such, considerable nanomedicine based therapies are undergoing clinical trials today [99].

One of challenges for nanoparticle-based therapy is to determine treatment efficacy using a model system that resembles patients’ tumor. PDX as a model meets this requirement. It has been demonstrated that PDX TNBC models are highly vascularized in comparison to cell line xenografts, resembling original patients’ tumors [100]. Although this field is advancing rapidly, specificity of nanoparticle-drug accumulation within TNBC PDX tumor as opposed to surrounding tissues and other organs due to enhanced permeability and retention effect has yet to be investigated. Using PDX model to determine the therapeutic efficacy of nanomedicine will provide novel, translatable and tangible approaches for the clinical treatment of TNBC patients.

**Figure 2:**
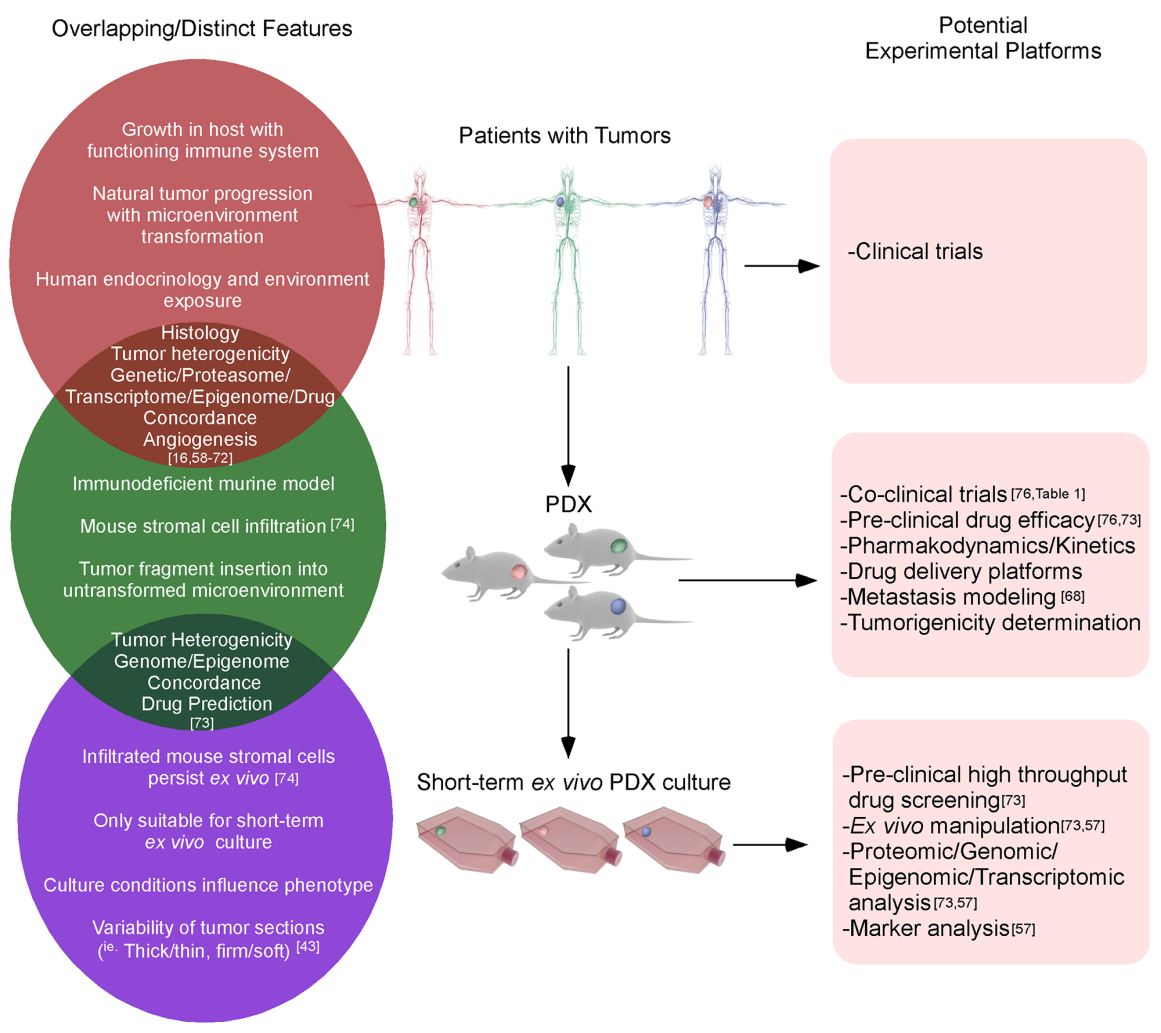
The similarities and differences between clinical, PDX and *ex vivo* cultured PDX tissues The growth of original tumors in patients will be influenced by the tumor microenvironment, immune system, *etc*. When the primary tumor tissues harvested from patients are inserted into immunodeficient mice, the majority of tumor properties could be retained. After *in vivo* passages, murine stromal cells could gradually infiltrate the tumor, although this will not significantly alter the tumor phenotypes until later passages. When PDX tissues are excised and cultured *ex vivo* for a short-term, genetic/epigenetic/drug predictions are still highly correlated with the original patients’ tumors.

To conclude, a hierarchy of patient tumors, *in vivo* PDX and short-term *ex vivo* cultured PDX tissues have been depicted with their respective overlapping or distinctive features (Figure 2). Since the development of PDX TNBC models is crucial for experimentation, we have included the procedures for the expansion/generation of PDX in NOD-SCID mice (Supplementary Materials and Supplementary Figure 1) [101].

## SUPPLEMENTARY MATERIALS FIGURES


