# Supplementary material for: Bridging the divide: preclinical research discrepancies between triple-negative breast cancer cell lines and patient tumors
Source: Oncotarget. 2017 Dec 4;8(68):113269–81. doi: 10.18632/oncotarget.22916 (PMC5762590; doi:10.18632/oncotarget.22916)
Supplement: Supplementary file 1 [file oncotarget-08-113269-s001.pdf]

Bridging the divide: preclinical research discrepancies between triple-negative breast cancer cell lines and patient tumors

SUPPLEMENTARY MATERIALS

PDX PROTOCOL

MATERIALS:

-Human TNBC primary tumor tissue (fresh attained from mouse or patient) or frozen (in DMEM media for primary tumor fragment culture + 10% DMSO)

-Ice

-6 well plate

-Scalpel

-Forceps

-1× phosphate-buffered saline (PBS)

-Scissors

-DMEM media for primary tumor fragment culture (DMEM/F12 media, 5% FBS, 1% penicillin/streptomycin, 1 μg/ml insulin, 0.5 ng/ml hydrocortisol and 3 ng/ml epidermal growth factor)

-Eppendorf Tubes

-Mr. Frosty freezing container

-NOD-SCID Mouse (Female)

-Isoflurane gas in oxygen and induction chamber/surgical platform

-Lidocaine/bupivacaine or approved alternative anesthetic

-Tear gel

-Insulin Needle

-Electric razor

-Wound clips

-Chlorhexidine antiseptic products

PROCEDURE:

1. Importantly, all steps should be carried out with maximum sterility and the fragments/tumors kept cold one ice when possible. Obtain patient tumor fragments either from the nitrogen freezer or freshly from either the patient or the mouse.

2. If the sample is fresh, extract the tumor in the fume hood using scalpels and scissors, remove hair and other tissue from the tumors. Afterwards place the whole tumors into a 6 well plate with 3mLs of DMEM media and remove any necrotic, whitish tissue. If the samples are frozen, thaw in a 37-degree water bath and transfer into a 6 well plate with 3 mls of DMEM media.

3. If dealing with a whole tumor, or large fragments, cut the sample into approximately 1mm x 1mm x 1mm chunks in the 6-well plate.

4. Once the tumor fragments for implantation are attained, fill the wells of a new 6 well plate with 2mLs of PBS and wash the samples by dropping them into each well, picking them up and redoing this throughout the 6-well plate. Once complete transfer, if looking to implant the fragments into NOD-SCID mice, place the fragments into an eppendorf tube with 1mL of DMEM/F12 empty media (without antibiotics, FBS or supplements) and proceed to step 6.

5. If freezing down cells, add 1 mL of freezing media (DMEM/F12, 5% FBS, 10% DMSO) to a cryovial tube and add up to 6 fragments per vial. Transfer vials to -80-degree freezer in a Mr. Frosty freezing container with isopropanol overnight and the next day transfer the vials to a nitrogen freezer. Importantly, when looking to thaw tumors, viability drops dramatically with each freeze and thaw cycle; hence, only take out the vials which are to be used and do not re-freeze tumors once thawed.

6. Prepare the surgery area for tumor implantation and ensure sterility of the area. Pick up NOD-SCID mice and inject Lidocaine/bupivacaine anesthetic mixture or suitable alternative via subcutaneous injection into the loose folds of skin on the neck area. Importantly, the NOD-SCID mice should only be operated on after at least a week to acclimate to its new environment.

7. Anesthetize the mice by exposing to 2% isoflurane gas in oxygen within an induction chamber. Once the mice are still and not twitching, add tear gel to the eyes and transfer the mouse to the surgical platform on its back and adjust the head so that a continuous flow of isoflurane gas can be delivered.

8. Shave the mice on the lower groin area, around the four bottom mammary fat pads. Remove any loose hair and disinfect the skin using chlorhexidine antiseptic products.

9. Make a small subcutaneous incision under the skin using scissors to expose the fat pad and using a different pair of scissors, insert the blades into the incision and using reverse-incision motions, expand the pocket moving towards the above mammary fad pad. Once close to the above mammary fat pad, remove the scissors and using sterile forceps, insert a tumor fragment into the pocket created and gently push the fragment as far up the pocket towards the above mammary fat pad as possible.

10. Once place properly, remove the forceps carefully without displacing the tumor fragment and seal the wound with a wound clip.

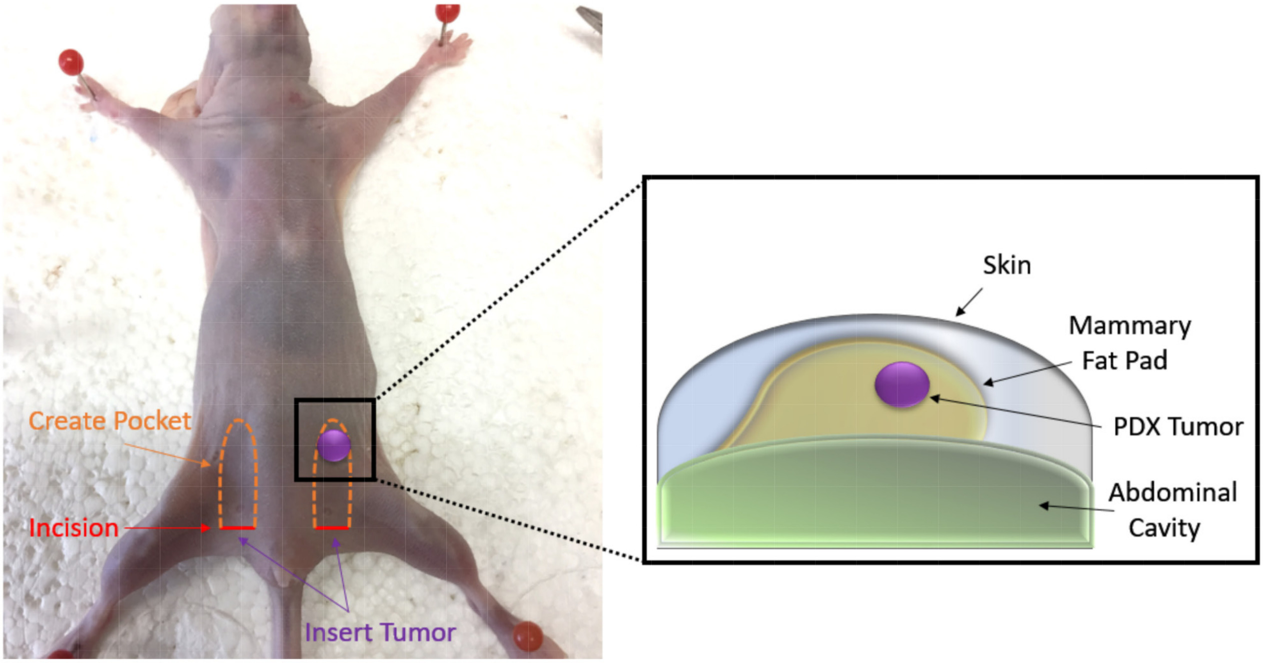

Supplementary Figure 1: Schematic of TNBC tumor fragment insertion procedure in NOD-SCID mice. An incision below last nipple is made and scissors is placed inside to expand the pocket between the skin and abdominal wall through inverse cutting motions. Once the pocket is large enough and around the above mammary fat pad, the tumor is inserted firmly to the end of the pocket and wound clips close the incision. To the right is an image of the environment the tumor is growing after the procedure.
